# The bidirectional gut microbiota-inflammation axis in gestational diabetes mellitus: pathogenesis, long-term outcomes, and therapeutic perspectives

**DOI:** 10.3389/fendo.2026.1902704

**Published:** 2026-08-03

**Authors:** Lei Xu, Yi Li, Yankun Feng, Yan Zhang, Jing Cai

**Affiliations:** 1 Department of Obstetrics and Gynecology, Wuhan Children’s Hospital Graduate Joint Training Base, School of Medicine, Wuhan University of Science and Technology, Wuhan, China; 2 Wuhan Children’s Hospital (Wuhan Maternal and Child Healthcare Hospital), Tongji Medical College of Huazhong University of Science and Technology, Wuhan, China; 3 School of Medicine JiangHan University, Wuhan, China

**Keywords:** gestational diabetes mellitus, gut microbiota, immune regulation, inflammatory cascade, maternal and infant health, metabolic disorder, probiotics

## Abstract

Gestational diabetes mellitus (GDM) is a highly prevalent metabolic disorder of pregnancy that carries immediate and long-term health risks for both mothers and their offspring. Emerging evidence implicates maternal gut microbiota dysbiosis as a critical contributor to the onset and progression of GDM, primarily through the induction of chronic low-grade systemic inflammation. This review synthesizes recent advances in understanding the compositional shifts in the maternal gut microbiota—including depletion of short-chain fatty acid–producing taxa such as *Bifidobacterium* and *Faecalibacterium*, and enrichment of pro-inflammatory genera—and examines how these changes compromise intestinal barrier integrity, promote microbial metabolite translocation, and subsequently activate innate immune pathways. The resultant elevation of pro-inflammatory cytokines directly impairs insulin signaling, exacerbating insulin resistance and fueling a vicious cycle between dysbiosis and metabolic deterioration. Beyond the peripartum period, this review highlights the emerging concept of transgenerational programming, whereby maternal GDM-associated dysbiosis and inflammation may shape the offspring’s microbiome, immune set-point, and metabolic trajectory, predisposing them to later-life metabolic and neurodevelopmental disorders. Finally, we critically evaluate current microbiota-targeted interventions, including probiotics, synbiotics, and dietary modifications, and discuss the sources of heterogeneity in clinical trial outcomes. By integrating these mechanistic and translational insights, this review proposes a conceptual framework for early risk stratification and personalized management of GDM, emphasizing that effective clinical translation requires moving beyond one-size-fits-all probiotic trials toward stratified, microbiome-guided strategies.

## 1 Introduction

Gestational diabetes mellitus (GDM) is clinically defined as glucose intolerance with first onset or recognition during pregnancy and has become a growing global public health concern (1). Its rising incidence imposes a considerable disease burden on both mothers and their offspring, owing to associated short- and long-term complications (2, 3). Although the precise etiology and pathophysiology of GDM remain incompletely understood, the condition is characterized by metabolic disturbances, including insulin resistance and chronic low-grade inflammation. GDM arises from a complex interplay between genetic predisposition and pregnancy-induced physiological changes, ultimately leading to impaired insulin sensitivity and disrupted maternal–fetal energy homeostasis (4, 5). Notably, approximately 50% of women with a history of GDM exhibit persistent postpartum glucose intolerance and face a markedly elevated risk—up to seven-fold—of progressing to type 2 diabetes mellitus (T2DM) in subsequent years (1). This progression underscores the urgent need to elucidate the fundamental mechanisms underlying GDM pathogenesis to inform more effective prevention and treatment strategies.

In recent years, the gut microbiota has been recognized as a key regulator of metabolic balance and immune function, with dysbiosis has been consistently associated with the development of various diabetic conditions, including T2DM and GDM (6). A growing body of evidence indicates that pregnant women with GDM harbor a distinct gut microbial community compared to normoglycemic controls, characterized by significant dysbiosis (7). This dysbiotic state is typically characterized by reduced microbial diversity and richness, decreased abundance of beneficial genera such as *Bifidobacterium*, *Lactobacillus*, and *Faecalibacterium*, and an increased prevalence of potentially pathogenic taxa, including *Enterococcus*, *Escherichia*, *Streptococcus*, and certain members of the *Ruminococcaceae* and *Prevotella families (*7, 8). These compositional changes can be detected as early as the first trimester, suggesting their potential value as early biomarkers for GDM risk. The gut microbial alterations observed in GDM resemble those identified in T2DM, pointing to shared pathological pathways involving metabolic and inflammatory dysregulation (1, 4). Furthermore, pregnancy itself induces substantial physiological adaptations, including hormonal changes and immune modulation, which profoundly shape the composition and functional capacity of the maternal gut microbiota (9). This creates a critical interface for host–microbe interactions that can influence the trajectory toward either metabolic health or disease.

The mechanistic link between gut dysbiosis and GDM development is increasingly attributed to impaired intestinal barrier function and the subsequent initiation of systemic inflammatory responses (10). Dysbiosis in GDM is associated with increased intestinal permeability, often referred to as “leaky gut”, as evidenced by elevated circulating levels of biomarkers such as zonulin and lipopolysaccharide (LPS) (11). LPS, an endotoxin component of Gram-negative bacterial cell walls, gains access to systemic circulation upon intestinal barrier failure. This translocation activates innate immune pathways, primarily through Toll-like receptor 4 (TLR4), leading to the secretion of pro-inflammatory cytokines, including tumor necrosis factor-alpha (TNF-α) and interleukin-6 (IL-6) (12). This persistent low-grade inflammatory state is a major driver of insulin resistance. Inflammatory cytokines disrupt insulin signaling cascades, such as the PI3K/AKT pathway, in peripheral tissues, thereby impairing glucose uptake and regulation (13). Concurrently, dysbiosis disturbs the synthesis of beneficial microbial metabolites, particularly short-chain fatty acids (SCFAs) such as butyrate, propionate, and acetate. These SCFAs are normally produced through the bacterial fermentation of dietary fiber by commensals including *Faecalibacterium* and *Lachnospiraceae (*7, 10). SCFAs serve as key signaling molecules that strengthen intestinal barrier integrity, attenuate inflammation, and enhance cellular insulin sensitivity. Their depletion in GDM therefore removes a protective metabolic and anti-inflammatory influence, further exacerbating insulin resistance and hyperglycemia (14).

**Figure 1:**
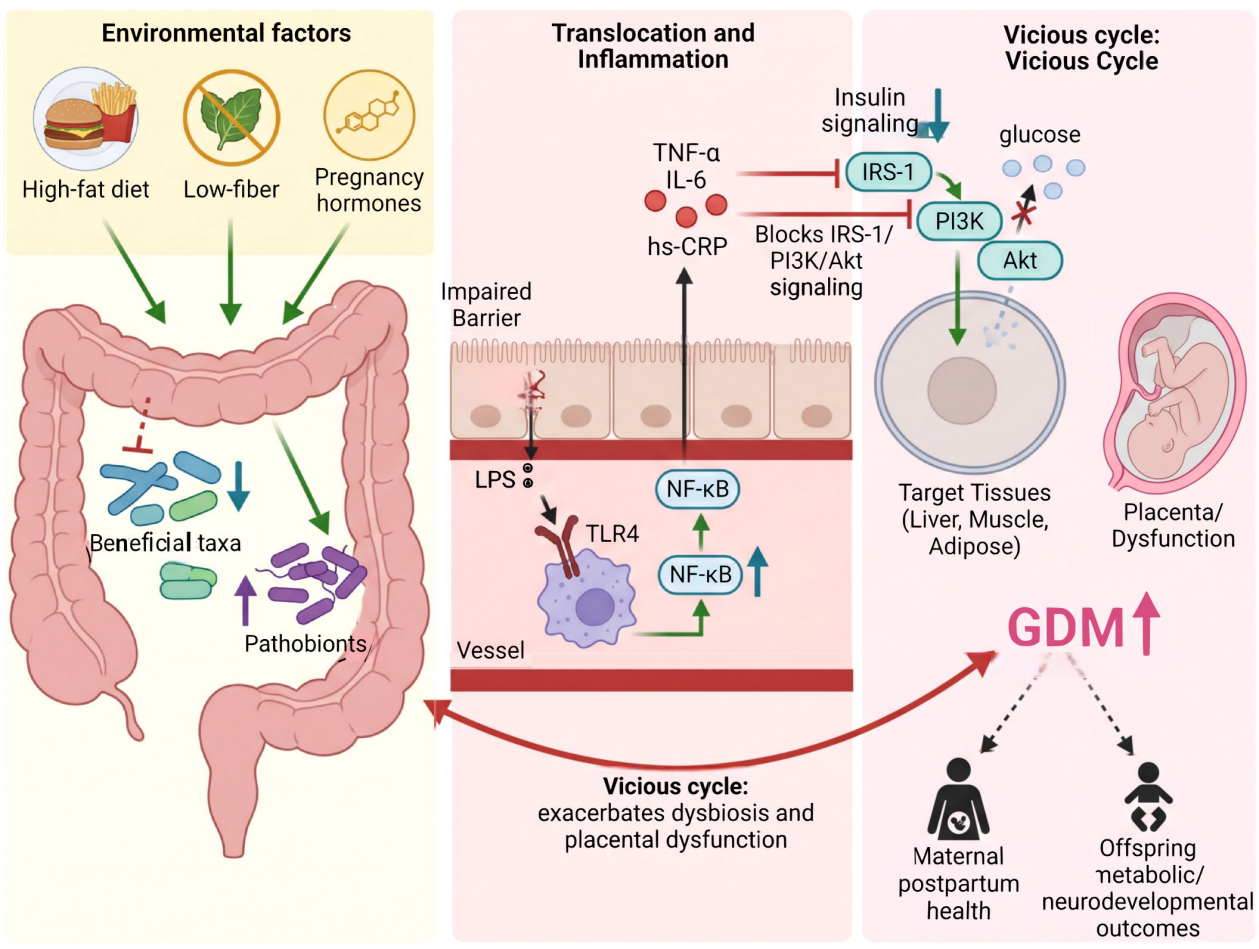
A conceptual framework illustrating the bidirectional interactions among environmental and physiological factors, gut microbiota dysbiosis, systemic low-grade inflammation, and the pathogenesis of GDM, with downstream consequences for both maternal and offspring health. The figure depicts three interconnected phases: (Left) Initiation phase – Environmental factors (e.g., high-fat diet, low-fiber, and pregnancy-associated hormonal) changes disrupt gut microbial ecology, reducing beneficial taxa (e.g., *Bifidobacterium*, *Lactobacillus*) while enriching potentially pathogenic bacteria, and compromising intestinal barrier integrity. (Center) Pro-inflammatory vicious cycle – Impaired barrier function facilitates translocation of bacterial components such as LPS into the circulation, activating innate immune receptors (e.g., TLR4/NF-κB pathway) and promoting a state of chronic low-grade inflammation characterized by elevated pro-inflammatory cytokines (TNF-α, IL-6, hs-CRP). These mediators directly interfere with insulin signaling, driving insulin resistance and ultimately GDM onset. (Right) Transgenerational cycle – Maternal hyperglycemia, insulin resistance, and systemic inflammation in GDM further perpetuate gut dysbiosis, placental dysfunction, and epigenetic modifications. Importantly, this phase highlights the “placenta–gut axis” as a bidirectional communication pathway through which maternal microbial metabolites and inflammatory signals modulate placental function, immune activity, and nutrient transfer, thereby affecting fetal growth and development. The vertical transfer of microorganisms and their metabolic byproducts shape the neonatal gut microbiota and influences long-term metabolic programming, increasing offspring risks of obesity, metabolic syndrome, and neurodevelopmental disorders. These three phases collectively constitute a self-reinforcing cycle, wherein gut dysbiosis and inflammation amplify each other across generations. Solid arrows represent well-established pathways; dashed arrows indicate mechanisms requiring further investigation.

This complex network of interactions extends beyond the maternal system, with significant implications for intergenerational health. Maternal gut dysbiosis and the associated pro-inflammatory and metabolic environment can influence fetal development through vertical transfer of microorganisms and their metabolic byproducts. This process may shape the composition of the neonatal gut microbiota and affect long-term metabolic programming. Offspring of mothers with GDM face an increased risk of developing obesity, metabolic syndrome, and neurodevelopmental disorders later in life (15, 16). This observation is consistent with the Developmental Origins of Health and Disease (DOHaD) hypothesis, originally proposed by Barker and colleagues, which posits that adverse exposures during critical windows of prenatal development—such as maternal undernutrition, protein restriction, or prenatal corticosteroid exposure—can program long-term metabolic and neurodevelopmental outcomes in both humans and experimental models (17–19). Therefore, while GDM represents one specific prenatal insult, it should be viewed within this broader conceptual framework. The placenta, a dynamic interface, is now recognized as part of a bidirectional communication pathway—the “placenta–gut axis”. Through this axis, maternal microbial metabolites and inflammatory signals can modulate placental function, immune activity, and nutrient transfer, thereby affecting fetal growth and development. This axis highlights the direct communication between maternal gut health and the fetal environment. Thus, the interconnected triad of GDM, gut microbiota dysbiosis, and inflammation constitutes a self-reinforcing cycle. This cycle not only drives maternal metabolic dysregulation but also exerts long-lasting effects on the offspring’s lifelong health trajectory. Consequently, elucidating this intricate network is essential for deepening our understanding of GDM pathogenesis and for identifying novel diagnostic and therapeutic strategies targeting the microbiota. This bidirectional relationship between maternal gut microbiota and host immunity constitutes a key pathway in GDM pathophysiology (as illustrated in **Figure 1**).

A comprehensive literature search was performed in PubMed, Web of Science, and Scopus databases for articles published up to May 2026. The search strategy combined terms related to ‘gestational diabetes mellitus’, ‘gut microbiota’, ‘inflammation’, ‘pregnancy’, and ‘therapeutic interventions’. We included original research articles (pro spective/retrospective cohorts, case-control studies, randomized controlled trials, and animal studies), systematic reviews, and meta-analyses published in English. Studies were excluded if they were conference abstracts, editorials, or case reports. Two independent reviewers (L.X. and Y.L.) screened titles/abstracts, and discrepancies were resolved by consensus with a third reviewer (Y.Z.). Although a formal risk-of-bias assessment was not performed for this narrative review, we have qualitatively discussed methodological limitations where relevant.

## 2 Maternal physiological remodeling and dynamic changes in gut microbiota during pregnancy

### 2.1 Impact of pregnancy hormones and metabolic changes on the gut microbiota

Pregnancy induces extensive physiological adaptations, primarily driven by significant shifts in hormonal and metabolic states, which collectively reshape the maternal gut microbial ecosystem. The marked increase in steroid hormones, particularly estrogen and progesterone, plays a central role in this transformation. Longitudinal studies in animal models, including wild baboons, indicate that transitions between reproductive states—especially into pregnancy—are associated with distinct changes in the composition and diversity of gut microbial communities (20). These hormonal fluctuations modify the intestinal environment through both direct and indirect mechanisms. For instance, variations in estrogen and progesterone levels strongly correlate with overall gut microbiota structure, although the specific microbial taxa involved vary depending on the reproductive phase (20). Estrogen and progesterone specifically can influence gut motility, gastric acid secretion, and bile acid metabolism, thereby creating conditions that favor the growth of particular bacterial groups. This bidirectional relationship is captured by the concept of the “microgenderome”, wherein sex steroids modulate gut microbiota composition—a dynamic that persists throughout pregnancy (21). The gut microbiota itself functions as an endocrine organ, interacting with hormones such as estrogen, androgens, and insulin, thereby contributing significantly to the female reproductive endocrine system across the lifespan (22). Concurrently, the metabolic profile of pregnancy undergoes a profound shift. The development of physiological insulin resistance and increased energy storage demands during mid-to-late gestation alters the maternal metabolic state. The gut microbiota adapts to these conditions, ostensibly to optimize energy harvest from dietary carbohydrates in support of fetal development. However, in some individuals, this adaptive process can become maladaptive, progressing into pathological dysbiosis. Such dysbiosis is implicated in the etiology of pregnancy-related disorders. In gestational diabetes mellitus (GDM), for example, alterations in the gut microbiota may contribute to disease development through mechanisms that include impaired intestinal barrier function and heightened intestinal energy balance (23). Notably, compositional differences in the gut microbiota that distinguish women who later develop GDM from healthy controls can be detected as early as the first or second trimester. This suggests that microbial shifts may precede the clinical manifestation of GDM, offering potential predictive value (24). The relationship is reciprocal: these sex steroid hormones and maternal metabolism influence the microbiota, while the microbiota, in turn, modulates maternal immune responses and metabolic processes, establishing a sophisticated regulatory framework critical for pregnancy outcomes (25). Consequently, the dynamic interplay among pregnancy-related hormonal changes, metabolic adjustments, and the gut microbial environment constitutes a fundamental axis that affects both maternal and fetal well-being.

### 2.2 Characteristic evolution of gut microbiota composition and diversity during pregnancy

The evolution of gut microbiota composition and diversity throughout a normal pregnancy follows a specific, dynamic trajectory that mirrors the physiological needs of gestation. From early to late pregnancy, the microbial community typically undergoes a decline in alpha diversity along with significant shifts in its overall structure (26). This progression is characterized by a transition toward a community profile associated with a pro-inflammatory state. A commonly reported change is an increased *Firmicutes/Bacteroidetes* ratio during mid-to-late pregnancy (23). Additionally, beneficial species such as *Akkermansia* tend to decrease in abundance, whereas the proportions of bacteria from phyla like *Pseudomonadota* (formerly *Proteobacteria*) and *Actinomycetota* (formerly *Actinobacteria*) may increase (26). These changes are not random but are closely synchronized with the host’s metabolic condition, forming an interconnected microbiota–metabolite regulatory system that supports pregnancy (27). In pregnancies complicated by gestational diabetes mellitus (GDM), this dysbiotic state is markedly intensified. The gut microbiota of women with GDM is characterized by a more pronounced decline in bacterial groups associated with metabolic health and anti-inflammatory properties. Specifically, there is a substantial reduction in butyrate-producing bacteria, including genera such as *Faecalibacterium* and *Roseburia (*28). Concurrently, a relative increase in the abundance of potential opportunistic pathogens, such as *Escherichia–Shigella* and members of the genus *Fusobacterium*, is frequently observed (29). This altered microbial composition compromises intestinal barrier integrity and metabolic stability. The inferred functional gene profiles of these microbial communities also differ; for instance, pathways related to amino sugar and nucleotide sugar metabolism are enriched in GDM patients (29). The functional consequences of these compositional changes are primarily mediated through alterations in microbial metabolites, which serve as key signaling molecules linking the gut microbiota to host physiology. Important metabolites include short-chain fatty acids (SCFAs) such as butyrate, whose levels decrease in dysbiosis and are critical for maintaining gut barrier function and immune regulation (30). Secondary bile acids, whose metabolism is heavily influenced by gut bacteria, also undergo changes, thereby affecting metabolic signaling pathways such as the farnesoid X receptor (FXR) axis (31). Moreover, elevated levels of microbial lipopolysaccharide (LPS) can contribute to systemic low-grade inflammation and insulin resistance—hallmarks of GDM (32). This intricate network of interactions underscores that the characteristic development of the gut microbiota during pregnancy is a precisely regulated process, and its disruption plays a central role in the pathophysiology of metabolic disorders such as GDM.

## 3 Mechanisms by which gut microbiota dysbiosis triggers inflammatory cascades during pregnancy

### 3.1 Impaired intestinal barrier integrity and metabolic endotoxemia

Gut microbiota dysbiosis is hypothesized to be a key contributor to the pathogenesis of gestational diabetes mellitus (GDM), primarily through its detrimental effects on intestinal barrier integrity. To systematically evaluate the evidence linking specific gut microbial taxa and their metabolites with GDM risk and inflammatory status, key studies are summarized in **Table 1**. This table provides an overview of prospective cohorts, cross-sectional comparisons, animal models, and intervention trials, highlighting the diversity of study designs and the consistent association between gut dysbiosis and metabolic impairment. This dysbiotic state is characterized by a marked reduction in beneficial bacterial populations, notably *Lactobacillus* and *Bifidobacterium*. These bacteria, frequently associated with secretory immunoglobulin A (SIgA), play an essential role in maintaining mucosal homeostasis. Conversely, there is an expansion of potentially pathogenic genera such as *Escherichia* and *Streptococcus (*33). This microbial imbalance leads to reduced expression of critical tight junction proteins, including zonula occludens-1 (ZO-1) and occludin, which are vital for preserving the paracellular barrier between intestinal epithelial cells (34, 35). Studies using GDM models have documented substantial decreases in these proteins, correlating with increased intestinal permeability, as reflected by elevated histological damage scores such as Chiu’s score (34, 35). The resulting barrier defect facilitates the translocation of microbial components from the gut lumen into the systemic circulation. A major consequence is the enhanced passage of lipopolysaccharide (LPS), a component of the outer membrane of Gram-negative bacteria, culminating in metabolic endotoxemia. Consistent findings show that serum LPS concentrations are significantly higher in women with GDM compared to normoglycemic pregnant controls (11, 33, 36). This process is driven by the “leaky gut” phenomenon, evidenced by positive correlations between markers of intestinal barrier impairment and blood glucose levels in GDM (11). The entry of LPS into the portal and subsequently systemic circulation acts as a primary trigger for inducing a state of chronic, low-grade systemic inflammation. Circulating LPS functions as a potent pathogen-associated molecular pattern (PAMP). It initiates inflammatory signaling by engaging the Toll-like receptor 4 (TLR4) complex on immune cells such as macrophages (37). This interaction activates downstream nuclear factor-kappa B (NF-κB) signaling pathways, promoting the transcription and secretion of pro-inflammatory cytokines. This mechanism establishes a direct link between gut-derived endotoxemia and the inflammatory cascade characteristic of GDM (37). Therapeutic strategies aimed at restoring gut barrier integrity—such as supplementation with xylooligosaccharides (XOS) or the probiotic *Akkermansia muciniphila*—have proven effective in upregulating ZO-1 and occludin expression, reducing intestinal permeability indices, and subsequently lowering circulating LPS levels in GDM models. These findings underscore the mechanistic importance of barrier dysfunction in the development of metabolic endotoxemia (34, 35).

**Table 1:** Summary of key gut microbiota and metabolite studies in GDM.

Study category	Population/model	Key microbiota/metabolite changes	Associated inflammatory/metabolic parameters	Key findings	Limitations
Prospective cohor (3)	Early-pregnancy Chinese women	↑Bacteroides; ↓Faecalibacterium	↑ Fasting glucose; ↑ inflammatory markers	Early-pregnancy gut microbiota alterations can predict subsequent GDM risk	Single ethnic population; limited generalizability; potential residual confounding from unmeasured dietary and lifestyle factors
Cross-sectional (32)	Second-trimester Shanghai Han women	↑Clostridium, Streptococcus; ↓Bifidobacterium, Lactobacillus	↑ HOMA-IR; ↑ serum endotoxin	GDM patients show reduced microbial diversity and enrichment of pro-inflammatory taxa	Cross-sectional design precludes causal inference; single time-point sampling; lack of longitudinal follow-up
Animal model (14)	High-fat diet-induced GDM mice	↓Akkermansia; ↑Lachnospiraceae	↑ Insulin resistance; ↑ placental inflammation	Fermentable fiber alleviates placental inflammation via Lachnospiraceae-derived butyrate	Animal model findings may not directly translate to humans; high-fat diet effects may confound microbiota-specific effects
Animal model (FMT) (88)	GDM mouse model (FMT from GDM patients)	Acquisition of GDM-associated microbiota	↓ Glucose tolerance; ↑ insulin resistance	Demonstration of causal transmission of GDM metabolic phenotype via gut microbiota	Gnotobiotic mouse model may not fully recapitulate human physiology; limited sample size; lack of long-term follow-up
Mechanistic study (11)	GDM patients vs. healthy controls	↓ SIgA-coated Lactobacillus reuteri	↑ Intestinal barrier damage	Depletion of L. reuteri is linked to GDM-related intestinal barrier dysfunction	Small sample size; cross-sectional design; mechanistic pathway not fully validated in human intervention
Intervention (RCT) (57)	GDM patients	Probiotic intervention (Lactobacillus + Bifidobacterium)	↓ FBG; ↓ HOMA-IR; ↓ hs-CRP	Specific probiotics improve glycemic control and inflammatory status	Short intervention duration; single probiotic formulation; lack of long-term maternal and offspring follow-up
Intervention (RCT) (62)	High-risk pregnant women	Multi-strain probiotics vs. placebo	No significant difference in GDM incidence or FBG	Mid-pregnancy probiotic initiation may be ineffective in certain high-risk populations	Late intervention window (mid-pregnancy); heterogeneous high-risk population; potential non-compliance; lack of microbiome profiling to confirm modulation

↑ indicates a statistically significant increase in the measured parameter, while ↓ indicates a statistically significant decrease, relative to the appropriate control group. FMT, Fecal Microbiota Transplantation; RTC, Randomized Controlled Trial.

### 3.2 Activation of immune cells and establishment of pro-inflammatory cytokine networks

The increased intestinal permeability observed in GDM facilitates the translocation of microbial products, most notably lipopolysaccharide (LPS), which acts as a potent activator of the innate immune system. Upon entering the circulation, LPS binds to TLR4 expressed on monocytes and macrophages residing in key metabolic tissues—including adipose tissue, the liver, skeletal muscle, and the placenta. This binding initiates intracellular signaling cascades that lead to NF-κB activation and the subsequent transcription and release of key pro-inflammatory cytokines (e.g., TNF-α, IL-6, IL-1β), thereby triggering localized and systemic inflammatory responses that directly impair insulin signaling in these tissues (12, 13). Consequently, both clinical studies and animal models of GDM consistently report significantly elevated concentrations of tumor necrosis factor-alpha (TNF-α), interleukin-6 (IL-6), and interleukin-1 beta (IL-1β) in serum and tissues. GDM rodent models, for instance, exhibit pronounced increases in IL-1β, IL-6, and TNF-α levels, which can be attenuated by interventions targeting the TLR4/NF-κB signaling axis. Similarly, GDM mice have been shown to have higher serum concentrations of TNF-α and IL-1β, reductions of which are achievable through treatments that enhance gut barrier function (34). This immune activation, driven by dysbiosis, extends its influence beyond innate immunity, disrupting the regulatory mechanisms of adaptive immunity, particularly those required for maintaining maternal-fetal immune tolerance.

## 4 The central role of inflammatory cascades in the pathogenesis of gestational diabetes mellitus

### 4.1 Inflammatory mediators disrupt insulin signaling pathways

Inflammatory mediators, particularly pro-inflammatory cytokines, are fundamental to the disruption of insulin signaling pathways—a key mechanism driving the insulin resistance characteristic of GDM. Key cytokines, including tumor necrosis factor-alpha (TNF-α) and interleukin-6 (IL-6), play a critical role in this process. These mediators interfere with the phosphorylation of insulin receptor substrate-1 (IRS-1), an essential initial step in insulin signal transduction. Specifically, TNF-α and IL-6 activate serine kinases that induce serine phosphorylation of IRS proteins, which antagonizes the tyrosine phosphorylation required for signal propagation (38). This aberrant modification effectively blocks downstream insulin signaling, thereby impairing insulin-stimulated glucose uptake into target cells.

Beyond direct interference with IRS-1, the inflammatory milieu triggers intracellular stress responses that further exacerbate insulin resistance. Prominent among these are the c-Jun N-terminal kinase (JNK) and the inhibitor of nuclear factor kappa-B kinase subunit beta/nuclear factor kappa-light-chain-enhancer of activated B cells (IKKβ/NF-κB) pathways (39). Activation of these pathways not only stimulates the production of additional inflammatory cytokines, establishing a self-perpetuating inflammatory cycle, but also directly impedes multiple stages of insulin action. Elevated circulating free fatty acids, common in GDM, can activate protein kinase C (PKC), which subsequently inhibits insulin signaling (39, 40).

The chronic low-grade inflammation associated with GDM also contributes to adipose tissue dysfunction. This is characterized by an altered secretion profile of adipokines, notably hyperleptinemia and hypoadiponectinemia. Hyperleptinemia can suppress insulin receptor expression via JAK/STAT signaling pathways, while reduced adiponectin levels diminish the activation of AMP-activated protein kinase (AMPK) in skeletal muscle and liver, further compromising insulin sensitivity. This subsequently impairs the translocation of glucose transporter type 4 (GLUT4) and subsequent glucose uptake. Additional adipokines, including chemerin, have been shown to disrupt insulin signaling cascades by targeting multiple proteins within the pathway and promoting inflammatory responses (39–41). The resultant imbalance in adipokine profiles exacerbates systemic insulin resistance and disrupts glucose and lipid metabolism, fostering a pathological milieu that perpetuates and amplifies insulin resistance (39). This complex interplay of cytokine activity, activation of intracellular stress pathways, and dysregulation of adipose tissue constitutes a central pathophysiological mechanism in GDM, directly linking inflammation to compromised metabolic homeostasis.

### 4.2 Inflammation and the self-perpetuating cycle of gestational metabolic dysfunction

In GDM, a self-reinforcing cycle emerges between metabolic dysregulation and inflammatory processes, accelerating disease progression and contributing substantially to adverse pregnancy outcomes. The GDM condition, characterized by hyperglycemia and dyslipidemia, inherently possesses pro-inflammatory properties. Elevated glucose and lipid levels can activate inflammatory pathways, such as the Toll-like receptor 4 (TLR4)/myeloid differentiation primary response 88 (MyD88)/NF-κB signaling axis, leading to increased production of cytokines including TNF-α, IL-6, and IL-1β (40). This establishes a deleterious feedback loop: hyperglycemia/hyperlipidemia → heightened inflammation → aggravated insulin resistance → further metabolic deterioration (40). This cycle is not merely theoretical but carries tangible clinical implications, accelerating GDM progression and substantially elevating the risk of associated complications, including gestational hypertension, preeclampsia, and fetal macrosomia (42). The inflammatory milieu contributes to endothelial dysfunction—a key factor in the vasculopathy observed in both preeclampsia and GDM—thereby linking maternal metabolic inflammation to adverse obstetric outcomes (42).

The clinical significance of this inflammatory cycle is underscored by the correlation between circulating inflammatory markers and disease severity. Concentrations of established markers, including C-reactive protein (CRP), IL-6, and TNF-α, are persistently elevated in women with GDM compared to those with normal glucose tolerance (43). These markers correlate not only with the severity of insulin resistance and hyperglycemia during gestation but also possess predictive value for postpartum metabolic health. Elevated CRP levels during pregnancy and the early postpartum period in women with GDM, for instance, predict an unfavorable cardiometabolic profile—including increased body weight, visceral adiposity, insulin resistance, and a higher incidence of metabolic syndrome one year after delivery—independently of body weight (44). Furthermore, nerve growth factor (NGF) levels are elevated in GDM patients and show a strong association with glucose metabolism, insulin resistance, and pancreatic β-cell function during the second trimester (45). The persistence of inflammation after delivery indicates that the inflammatory insult associated with GDM has long-term consequences for maternal health, potentially explaining the elevated lifetime risk of type 2 diabetes and cardiovascular disease in these women (44, 45). Consequently, the inflammatory–metabolic vicious cycle in GDM constitutes a key driver of both acute pregnancy complications and long-term maternal metabolic sequelae.

## 5 Long-term consequences of the maternal microbiota-inflammation axis on offspring health

### 5.1 The intrauterine environment and vertical transmission of microbiota

Accumulating evidence challenges the traditional view of a sterile intrauterine environment, suggesting that maternal gut microbiota or their metabolic byproducts may reach the intrauterine compartment, potentially crossing the placental barrier and establishing a distinct intrauterine microbiome (46). This notion is supported by metagenomic studies in non-human primates. In animal models, such as Rhesus macaques, significant microbial transfer occurs between mother and fetus. Microbial communities found in the placenta and umbilical cord more closely resemble those of fetal organs than the maternal gut microbiota (47), indicating a specialized or adapted microbial presence at the maternal–fetal interface. Human studies have identified microbial communities in placental tissue, amniotic membranes, and umbilical cord blood, providing direct evidence that the uterine environment is not sterile and that these placental tissues serve as important sources for the initial microbial colonization of the infant’s gastrointestinal tract (48). The underlying mechanisms likely involve the transfer of microbial components or metabolites through maternal circulation. Although the placenta acts as an effective barrier against pathogens, it may permit the passage of microbial signals that influence the fetal milieu (49). This vertical transmission is essential for the initial development and programming of the fetal immune system. Exposure to a maternal dysbiotic microbiota and its associated inflammatory environment, as may occur in GDM, can potentially affect fetal immune maturation, predisposing the offspring toward a pro-inflammatory state. The observed convergence between maternal and fetal microbial profiles, alongside distinct differences from adult microbiota, underscores the unique nature of the fetal microbial environment and its potential susceptibility to maternal disturbances (47). Furthermore, the fetal microbiota exhibits functional distinctions—such as a less developed capacity for carbohydrate metabolism compared to adults—and shares antibiotic resistance genes with the maternal microbiome, indicating potential routes for vertical transfer of functional characteristics (47). Therefore, the intrauterine period represents a foundational stage in which maternal microbial and metabolic signals contribute to fetal immune programming, and dysregulation in conditions such as GDM poses a risk of altering this critical developmental pathway.

### 5.2 Potential programming effects on offspring metabolism and neurodevelopment

The early life period, encompassing both prenatal and neonatal stages, represents a critical window for programming metabolic and immune systems, thereby establishing long-term health trajectories. Maternal conditions such as GDM, accompanied by dysregulation of the microbiota–inflammation axis, may elevate the long-term risk for offspring developing obesity, type 2 diabetes, allergic diseases, and neurodevelopmental disorders, potentially through mechanisms including epigenetic modifications. Prenatal exposure to environmental stressors such as endocrine-disrupting chemicals, which can disturb maternal metabolic and microbial homeostasis, has been investigated for its effects on fetal development and remains an area requiring further research into its programming consequences (50). Beyond environmental chemicals, pharmacological agents represent another category of prenatal exposures with the capacity to induce long-term metabolic programming in offspring. Prenatal exposure to the synthetic glucocorticoid dexamethasone—used clinically to accelerate fetal lung maturation—has been shown to increase hepatic triglyceride accumulation, exacerbate hepatic steatosis, intensify alterations in hepatocarcinogenesis markers, program pancreatic β- and α-cell development, and promote intestinal alterations in offspring (19, 51–53). These findings highlight that diverse prenatal insults—including both chemical and pharmacological agents—can converge on common pathways of developmental programming, thereby predisposing offspring to metabolic disease. Experimental evidence from animal models demonstrates that maternal exposure to compounds such as di-(2-ethylhexyl)-phthalate (DEHP) impairs maternal glucose metabolism and placental function, alters the expression of key genes in offspring livers, and results in metabolic disorders such as hyperglycemia in young offspring (54). This metabolic programming is mechanistically linked to the vertical transmission of a dysbiotic gut microbiota from mother to offspring—wherein dominant microbial phyla are altered—and to changes in placental signaling pathways, such as the mTOR pathway, which is critical for nutrient sensing and fetal growth (54, 55). The early colonization pattern of the offspring’s gut microbiota is significantly shaped by the maternal microbiota, involving vertical transmission from multiple maternal sites including the gut, placental tissues, skin, and breast milk, with taxa such as *Bifidobacterium* and *Lactobacillus* serving as pivotal contributors (46, 48). This initial colonization constitutes a dynamic, multi-site process influenced by variables including mode of delivery and infant feeding practices. Disturbances in this process, related to cesarean delivery, formula feeding, or antibiotic administration, have been linked to persistent alterations in microbial composition. Such deviations in the early establishment of the microbiota may extend throughout childhood, forming a biological basis for heightened risk of long-term health conditions. Compromised immune system development resulting from early dysbiosis, for instance, correlates with an increased susceptibility to inflammatory and neurodevelopmental disorders (46). Longitudinal cohort studies, including research on HIV and antiretroviral therapy exposure, aim to elucidate how perinatal exposures contribute to immune–metabolic dysfunction and adverse outcomes such as growth impairment and compromised neurocognitive development (56). These investigations underscore the intricate network of factors that shape offspring health trajectories. Consequently, the maternal microbial and inflammatory environment during the perinatal period serves as a crucial programming signal. Dysbiosis during this phase may establish a durable metabolic and immunological signature that increases the offspring’s predisposition to chronic diseases later in life.

## 6 Intervention strategies for GDM and future research directions involving gut microbiota modulation

### 6.1 Clinical investigations of probiotics, prebiotics, and synbiotics

Numerous clinical trials have assessed the efficacy of specific probiotic strains in preventing and managing GDM, yet the evidence presents a nuanced and often inconsistent picture. Several meta-analyses and systematic reviews have concluded that probiotic supplementation can offer benefits for glycemic regulation and inflammatory parameters in women with GDM. A meta-analysis of randomized controlled trials (RCTs) demonstrated that probiotic intake significantly lowered fasting plasma glucose by an average of approximately 3.10 mg/dL (57). Another extensive umbrella meta-analysis, encompassing data from 33,378 participants, reported that probiotic supplementation resulted in notable reductions in fasting blood glucose (FBG), fasting insulin levels, and the Homeostatic Model Assessment of Insulin Resistance (HOMA-IR) score (58). Documented benefits include decreases in inflammatory markers such as high-sensitivity C-reactive protein (hs-CRP), interleukin-6 (IL-6), and tumor necrosis factor-alpha (TNF-α), alongside reductions in oxidative stress indicators like malondialdehyde (MDA) (59). Interventions with probiotics, particularly formulations containing *Lactobacillus* and *Bifidobacterium* species, have been correlated with enhanced insulin sensitivity, supported by observed increases in the Quantitative Insulin Sensitivity Check Index (QUICKI) and decreases in HOMA-IR values (60, 61). These metabolic and anti-inflammatory effects are believed to be mediated through modification of gut microbiota composition, increased production of short-chain fatty acids (SCFAs), and subsequent activation of signaling pathways involving G-protein coupled receptors (GPCRs), which stimulate glucagon-like peptide-1 (GLP-1) secretion (60). The metabolic benefits of probiotics are not restricted to GLP-1 secretion. Gut-derived hormones such as glucose-dependent insulinotropic polypeptide (GIP), cholecystokinin (CCK), and fibroblast growth factor 15/19 (FGF15/19 in rodents/humans) also play critical roles in glucose homeostasis, insulin secretion, and energy balance. For example, GIP potentiates insulin secretion in a glucose-dependent manner, CCK regulates satiety and pancreatic enzyme secretion, and FGF15/19 modulates bile acid synthesis and hepatic glucose metabolism. Probiotic-induced changes in the gut microbiota can influence the secretion of these hormones through modulation of the enteroendocrine cell environment and bile acid profiles. A comprehensive understanding of these multi-hormonal effects is essential for evaluating the full therapeutic potential of probiotics in GDM. Furthermore, probiotic or synbiotic supplementation has been associated with improved neonatal outcomes, including a lower incidence of macrosomia and reduced rates of newborn hospitalization (57, 59). However, not all research supports these favorable results. A large-scale, double-blind, placebo-controlled RCT conducted in a high-risk obstetric population found that a multi-strain probiotic supplement initiated in the second trimester did not reduce the incidence of GDM or improve fasting glucose levels compared to placebo (62). Similarly, a Cochrane review and other meta-analyses have indicated that the evidence for probiotics affecting GDM risk or maternal outcomes such as hypertensive disorders is of low certainty, highlighting considerable heterogeneity and wide confidence intervals in the pooled findings (63, 64). These discrepancies underscore the significant impact of various intervention parameters. Subgroup analyses indicate that factors such as intervention duration, specific strains and dosage of probiotics, implementation of a pre-intervention washout phase, and concurrent dietary modifications may substantially influence the effectiveness of probiotic supplementation (65). Interventions involving physical activity, for instance, demonstrated greater efficacy in lowering GDM risk among participants with a normal body mass index compared to those with obesity, underscoring the importance of individual characteristics.

Prebiotics—defined as non-digestible dietary components that selectively promote the proliferation of beneficial bacteria—provide an indirect strategy for modulating the gut ecosystem and immune function. Investigations into prebiotics such as galactooligosaccharides (GOS) and inulin-type fructans (ITF) in the context of GDM are increasingly emerging. An initial pilot randomized controlled trial indicated that GOS supplementation during pregnancy was both safe and well-tolerated. Although it did not lead to significant changes in glucose or lipid metabolism parameters, it induced specific shifts in gut microbiota composition, including an increased abundance of *Paraprevotella* and *Dorea (*66). Specific bacterial genera have been associated with distinct metabolic benefits. *Bifidobacterium* and *Lactobacillus* produce lactate and acetate, which lower intestinal pH, inhibit pathogenic bacteria, and enhance intestinal barrier function (7). *Faecalibacterium prausnitzii*, a major butyrate producer, provides energy for colonocytes, strengthens tight junctions, and exerts anti-inflammatory effects via NF-κB inhibition (10). *Akkermansia muciniphila* reinforces the mucus layer, reduces endotoxemia, and improves insulin sensitivity. The depletion of these beneficial genera in GDM removes these protective functions, thereby contributing to metabolic deterioration. A more recent study reported that GOS targeted the gut microbiota to enhance hexanoic acid production—a short-chain fatty acid that exhibited a negative correlation with total cholesterol and LDL levels—suggesting a potential favorable effect on lipid metabolism, particularly in overweight and obese individuals with GDM (67). Animal models support this mechanistic insight; ITF administration in a high-fat diet-induced GDM mouse model improved glucose and lipid metabolism disorders, elevated gut microbial diversity, and enriched beneficial genera such as *Bifidobacterium* and *Akkermansia*(68). Furthermore, administration of a prebiotic phytocompound (green leaf extract) in a GDM mouse model not only improved metabolic parameters but also reversed gut dysbiosis, enhanced gut barrier integrity, and mitigated behavioral and cognitive deficits in offspring, outperforming metformin in certain aspects (69). These observations demonstrate how prebiotics can elicit systemic anti-inflammatory and metabolic benefits by cultivating a healthier gut microbiota and boosting the synthesis of beneficial metabolites such as short-chain fatty acids.

Synbiotics, which integrate probiotics with prebiotics, are designed to synergistically improve the viability and colonization of live microbial supplements within the gastrointestinal tract. This combined approach is regarded as a potentially more potent interventional strategy. Clinical trials have demonstrated that synbiotic supplementation in women with GDM can result in marked improvements in glycemic control and lipid profiles. Synbiotics have been shown to reduce fasting glucose, insulin concentrations, HOMA-IR, and triglyceride levels while augmenting insulin sensitivity as measured by QUICKI (70, 71). A randomized controlled trial combining probiotics with selenium also documented beneficial effects on glycemic status, lipid profiles, and gene expression of peroxisome proliferator-activated receptor gamma (PPAR-γ) (72). Another investigation found that a 6-week synbiotic intervention significantly improved the logTG/HDL-C ratio—an atherogenic index—indicating a potential role in reducing future cardiovascular disease risk linked to GDM (73). The proposed mechanisms underlying synbiotic efficacy are multifaceted, encompassing modulation of gut microbiota composition, regulation of microbial metabolites, enhancement of intestinal barrier function, and reduction of systemic inflammation and oxidative stress (74). Despite this promising outlook, significant challenges persist in optimizing synbiotic interventions. The ideal selection of specific probiotic strains, their synergistic prebiotic counterparts, effective dosage, and precise timing of intervention remain unstandardized and warrant further investigation (75, 76). Heterogeneity in study designs, probiotic formulations, and participant characteristics contributes to the inconsistent outcomes observed across the literature.

**Figure 2:**
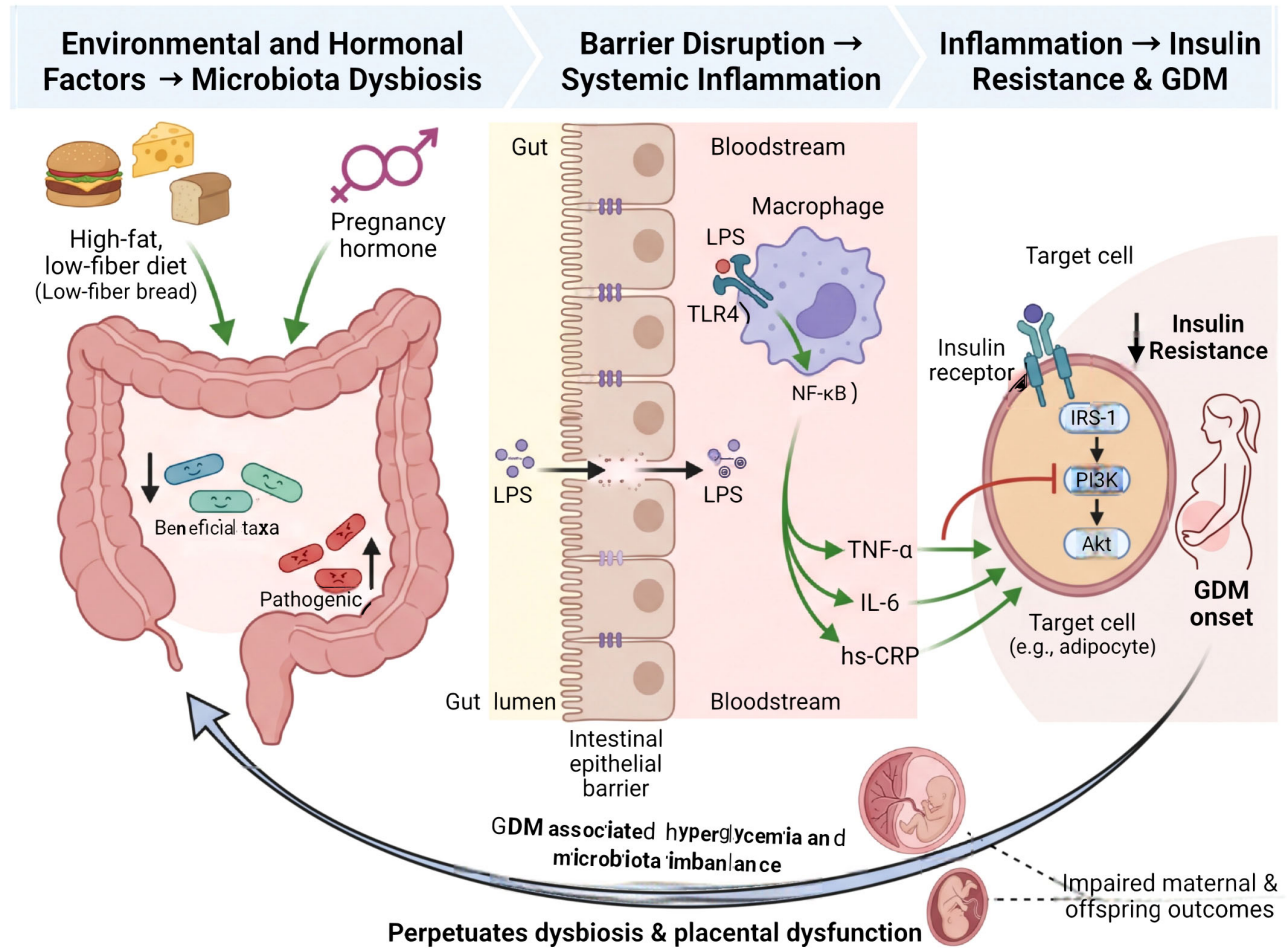
A conceptual model linking gut microbiota dysbiosis to GDM. The figure depicts the mechanistic cascade from environmental and hormonal triggers to GDM onset, emphasizing the self-reinforcing gut microbiota–inflammation axis. (Left) Environmental and hormonal factors drive microbiota dysbiosis: A high-fat, low-fiber diet and pregnancy-related hormonal changes disrupt gut microbial homeostasis, reducing beneficial taxa (e.g., *Bifidobacterium*, *Faecalibacterium*, *Akkermansia*) and enriching pathogenic species, leading to dysbiosis. (Center) Barrier disruption and systemic inflammation: Dysbiosis impairs intestinal epithelial barrier integrity (reduced tight junction proteins ZO-1, occludin), facilitating LPS translocation into circulation. LPS activates TLR4 on macrophages, triggering NF-κB signaling and release of pro-inflammatory cytokines (TNF-α, IL-6, hs-CRP), resulting in chronic low-grade systemic inflammation. (Right) Inflammation drives insulin resistance and GDM onset: Pro-inflammatory cytokines impair IRS-1 phosphorylation and PI3K/Akt signaling in target tissues (adipose, liver, skeletal muscle), causing peripheral insulin resistance—a key driver of GDM. GDM-induced metabolic disturbances further exacerbate dysbiosis and placental dysfunction, perpetuating inflammation and insulin resistance in a vicious cycle.

The existing body of intervention research presents mixed findings, underscoring the critical need for enhanced accuracy in microbiota characterization and the development of tailored intervention approaches. The paradigm of precision prevention is increasingly recognized, wherein an individual’s specific attributes—including baseline gut microbiota profile, body mass index, polycystic ovary syndrome (PCOS) status, and metabolic parameters—influence therapeutic response (65). Future investigations should move beyond generalized methodologies. There is an urgent need for large-scale, rigorously designed randomized controlled trials that not only evaluate clinical endpoints but also incorporate comprehensive multi-omics analyses. Such studies are essential for identifying distinct microbial markers or metabolic networks predictive of GDM risk and therapeutic response to probiotics, prebiotics, or synbiotics (77) This approach will facilitate the development of targeted, evidence-based guidelines for specific demographic subgroups, thereby advancing more effective and personalized strategies for GDM prevention and management through gut microbiota modulation. Heterogeneity in study designs, probiotic formulations, and participant characteristics contributes to the inconsistent outcomes observed across the literature (**Figure 2**).

### 6.2 Dietary and lifestyle interventions from a microbiome viewpoint

A diet rich in dietary fiber is fundamental to GDM management, and its beneficial effects can be comprehensively understood through gut microbiota mechanisms. High-fiber dietary patterns, such as the Mediterranean diet or the Dietary Approaches to Stop Hypertension (DASH) diet, demonstrate an inverse correlation with GDM incidence (78). These nutritional patterns promote the proliferation of beneficial commensal bacteria that ferment non-digestible fibers. The primary metabolites of this fermentation are short-chain fatty acids (SCFAs), including acetate, propionate, and butyrate. SCFAs serve a vital dual function in GDM management: they exert significant anti-inflammatory effects and enhance insulin sensitivity. Notably, butyrate acts as the main energy substrate for colonocytes, strengthening the intestinal epithelial barrier and reducing systemic dissemination of pro-inflammatory agents such as LPS (79). Propionate and acetate participate in gluconeogenesis and lipid metabolism, thereby regulating host energy balance. SCFAs activate G-protein coupled receptors (GPRs) such as GPR41 and GPR43 on intestinal enteroendocrine L-cells, stimulating the release of glucagon-like peptide-1 (GLP-1) and peptide YY (PYY). GLP-1 enhances glucose-stimulated insulin secretion, suppresses glucagon release, and induces satiety, collectively improving glycemic regulation (60). Furthermore, SCFAs can regulate immune cell activity and suppress the NF-κB pathway—a central mediator of inflammatory responses (80). Consequently, the microbial conversion of dietary fiber into SCFAs constitutes a core mechanistic link between high-fiber intake, attenuated systemic inflammation, enhanced insulin sensitivity, and improved glucose homeostasis, providing a robust scientific rationale for this conventional dietary strategy in GDM (81). Similarly, dietary interventions such as high-fiber or Mediterranean-style diets can enhance the secretion of multiple gut hormones beyond GLP-1. Increased SCFA production from fiber fermentation stimulates L-cells to release GLP-1 and peptide YY (PYY), while also influencing K-cells to secrete GIP and I-cells to secrete CCK. Furthermore, SCFAs can modulate FGF15/19 expression in the ileum, thereby impacting bile acid metabolism and hepatic insulin sensitivity (14). Thus, the beneficial effects of dietary interventions on glycemic control in GDM are likely mediated through a coordinated hormonal response involving GIP, CCK, and FGF15/19, in addition to GLP-1.

Importantly, dietary interventions may also directly benefit pancreatic β-cell function, which is critical given that impaired insulin secretion is a hallmark of GDM. SCFAs, particularly butyrate and propionate, have been shown to stimulate GLP-1 secretion from intestinal L-cells, which in turn enhances glucose-stimulated insulin secretion from pancreatic β-cells via the GLP-1 receptor (14). Additionally, SCFAs can directly act on β-cells through free fatty acid receptors (FFAR2 and FFAR3), potentiating insulin release in a glucose-dependent manner (10). In animal models, high-fiber diets have been reported to increase β-cell mass and improve glucose tolerance, effects that are mediated by the gut microbiota–SCFA axis. Furthermore, polyphenols found in Mediterranean diets may protect β-cells from oxidative stress and apoptosis (79). Thus, dietary interventions improve not only peripheral insulin sensitivity but also β-cell secretory capacity, addressing both arms of the insulin–glucose axis. These effects occur primarily in the pancreas (β-cells) and the intestine (L-cells), with secondary benefits in the liver and adipose tissue.

Regular physical activity during pregnancy is another well-established intervention that enhances metabolic health, with its benefits partially mediated through gut microbiota alterations. Exercise has been shown to increase microbial diversity and modify gut microbiota composition across various populations. In the context of pregnancy and GDM, physical activity can mitigate the dysbiosis frequently associated with metabolic impairment. Although direct human studies elucidating exercise-induced microbiome changes specifically in GDM are still emerging, the general mechanisms provide valuable insights. Exercise can shorten intestinal transit time, modify bile acid metabolism, and lower systemic inflammation—all of which influence the microbial ecosystem (82). A primary outcome is the reduction of chronic low-grade inflammation. Physical activity decreases concentrations of pro-inflammatory cytokines such as TNF-α and IL-6, which are commonly elevated in insulin-resistant conditions such as GDM (73). By enhancing gut barrier integrity and reducing inflammatory signaling, exercise fosters an environment less conducive to metabolic dysfunction. Physical activity also promotes the proliferation of beneficial symbiotic bacteria that produce metabolites such as SCFAs, while reducing the abundance of pro-inflammatory bacteria. A systematic review indicated that physical exercise interventions lead to a more significant reduction in GDM risk in individuals with a normal body mass index (BMI) compared to those with obesity, suggesting that the metabolic benefits of exercise—including its potential effects on the microbiome—may be influenced by baseline adiposity (65). Therefore, from a microbiome perspective, regular physical activity during pregnancy likely contributes to GDM management by promoting a more diverse and stable gut microbiota, increasing the production of anti-inflammatory and insulin-sensitizing microbial metabolites, and disrupting the cycle of inflammation and insulin resistance.

Understanding the mechanistic role of the gut microbiota in mediating the effects of dietary and exercise interventions provides a more robust and integrated scientific basis for advocating these lifestyle modifications. This shifts the focus from merely recommending generic diet or exercise to promoting specific dietary components and activity patterns that actively cultivate a health-promoting microbial environment. Such an approach can improve patient education and adherence, as the recommendations are grounded in a clear biological pathway linking lifestyle choices to metabolic outcomes through the microbiome. The “Healthy Gut Diet” intervention, for instance—developed based on the experiences of women with GDM and incorporating behavior change techniques—resulted in sustained postpartum improvements in diet quality, fiber intake, and micronutrient status compared to standard care (83). This demonstrates that well-designed, microbiome-informed antenatal interventions can yield long-term benefits. Moreover, this mechanistic understanding paves the way for combined or sequential interventions. A dietary intervention that enhances SCFA production, for example, might prepare the gut environment to increase the efficacy of subsequent probiotic supplementation. Future research should continue to elucidate these microbiome-mediated pathways, employing multi-omics approaches to monitor how specific dietary components and exercise regimens modify microbial taxonomy, gene expression, and metabolic output in pregnant women. This will advance the field from associative to causal explanations for the effectiveness of these fundamental GDM management strategies.

### 6.3 Emerging microbiome-based approaches: postbiotics, precision nutrition, and multi-omics

Beyond traditional probiotics and prebiotics, several novel strategies are gaining traction in the field of GDM management. Postbiotics—bioactive microbial metabolites including SCFAs, secondary bile acids, and exopolysaccharides—represent a promising approach for directly delivering anti-inflammatory and insulin-sensitizing signals without the need for live microorganisms. Oral administration of butyrate or propionate, for example, has been shown to improve insulin sensitivity and reduce inflammation in animal models of metabolic disease (6, 10, 14).

Precision nutrition, guided by individual gut microbiota profiles, is another emerging paradigm. Rather than applying a one-size-fits-all dietary or probiotic intervention, this approach tailors dietary recommendations—such as specific fiber types or polyphenol-rich foods—to an individual’s baseline microbial composition and metabolic needs (34) Early studies in type 2 diabetes have demonstrated that personalized dietary interventions based on gut microbiota data can significantly improve glycemic control, and similar strategies are now being explored in the context of GDM.

Multi-omics approaches—integrating metagenomics, metabolomics, proteomics, and transcriptomics—offer a systems-level understanding of host–microbe interactions in GDM. By simultaneously profiling the gut microbiota, circulating metabolites, and host gene expression, these approaches can identify causal pathways and discover novel biomarkers for early risk stratification. Integrated metagenomic and metabolomic analyses have revealed that specific microbial species and their metabolic products—such as imidazole propionate and branched-chain amino acids—are associated with insulin resistance in GDM. The application of multi-omics in large, well-phenotyped cohorts will be critical for advancing precision medicine in this field.

### 6.4 Future research directions and technical challenges

A critical priority for future research is to launch large-scale, prospective cohort studies beginning in the pre-conception period or early pregnancy. Such studies are essential to establish causality and identify specific microbial biomarkers that can reliably predict the subsequent onset of GDM. Current evidence indicates that alterations in the gut microbiota are present in women who later develop GDM, even during early pregnancy (84). However, most existing studies are cross-sectional or have limited sample sizes, restricting their predictive capacity. Large prospective cohorts with repeated biosampling are necessary to characterize the temporal dynamics of the gut microbiota throughout gestation. These studies should aim to identify a “gut microbiota signature”—a specific configuration of bacterial taxa, their functional genes, or derived metabolites—associated with a high risk of GDM. Validating such signatures across diverse populations will be essential for developing non-invasive, microbiome-based predictive tools.

Another fundamental area requiring in-depth investigation is the mechanistic role of specific gut microbiota-derived metabolites within the inflammatory and metabolic pathways linked to GDM. Beyond the extensively studied SCFAs, metabolites such as secondary bile acids and trimethylamine-N-oxide (TMAO) require dedicated investigation. Bile acids function as crucial signaling molecules that activate receptors including the farnesoid X receptor (FXR) and Takeda G protein-coupled receptor 5 (TGR5), which regulate glucose, lipid, and energy homeostasis (85, 86). Gut microbiota extensively modify primary bile acids into secondary forms, a process that modulates their signaling efficacy, and dysbiosis in GDM may alter the composition of the bile acid pool, thereby contributing to metabolic dysregulation. Similarly, TMAO, a microbial metabolite produced from dietary precursors like choline and carnitine, has been linked to inflammation, insulin resistance, and cardiovascular disease in non-pregnant individuals (87); however, its role in GDM pathophysiology remains largely undefined. Future research should employ targeted metabolomic approaches to quantify these and other relevant metabolites in longitudinal samples. Integrating metabolomic profiles with metagenomic and host transcriptomic data will facilitate the construction of detailed mechanistic networks, moving from correlative observations to establishing causation. Animal models, including germ-free or gnotobiotic mice colonized with human microbiota, will be essential for experimentally validating these hypothesized causal relationships.

A further promising avenue involves exploring more direct microbiota-targeted interventions, such as fecal microbiota transplantation (FMT), in animal models to evaluate the therapeutic potential of modifying the gut ecosystem. While probiotics and prebiotics aim to modulate the existing microbial community, FMT involves the transfer of an entire microbial consortium from a healthy donor. Before any consideration of human trials during pregnancy—which would involve substantial ethical and safety considerations—rigorous preclinical studies in GDM animal models are imperative. Research should assess whether FMT from healthy pregnant donors can prevent or reverse GDM-related phenotypes in recipient animals (88). These models also provide an opportunity to investigate safety profiles, including risks associated with pathogen transfer or ecological destabilization. Concurrently, research into next-generation probiotics and postbiotics holds promise. For example, pasteurized *Akkermansia muciniphila* has been shown to improve glucose intolerance and placental inflammation in a GDM mouse model, with its outer membrane protein Amuc_1100 identified as a key active component (89).

To translate these mechanistic insights into clinical practice, several key avenues require further investigation. First, longitudinal studies characterizing the gut microbiota from the periconceptional period through postpartum are needed to identify critical windows for intervention. Second, advanced gnotobiotic models and well-controlled human trials are essential to establish causality for specific microbial strains and metabolites. Third, the efficacy of microbiome-targeted interventions should be assessed not only by maternal glycemic control but also by long-term offspring outcomes, including the incidence of obesity, type 2 diabetes, and neurodevelopmental disorders. Finally, the development of precision nutrition approaches—guided by individual microbiome profiles, dietary habits, and genetic background—will be crucial for maximizing therapeutic benefit and minimizing heterogeneity in clinical outcomes.

Ultimately, addressing these research challenges requires the integration of multi-omics technologies—including metagenomics, metatranscriptomics, metabolomics, and proteomics—applied to well-characterized cohorts and intervention studies. This systems biology approach is crucial for advancing beyond mere taxonomic descriptions toward understanding functional alterations within microbial communities and their causal connections to host metabolic states, thereby translating microbiome research into tailored strategies for predicting, preventing, and managing GDM.

### 6.5 Methodological considerations and limitations of current microbiome studies

Despite the compelling evidence linking gut microbiota dysbiosis to GDM, several methodological limitations warrant careful consideration when interpreting the available literature. Variability in the diagnostic criteria for GDM across studies—including differences in glucose load (75 g versus 100 g), diagnostic thresholds (such as those defined by IADPSG, Carpenter-Coustan, or WHO), and the timing of screening—contributes to heterogeneity in study populations and complicates cross-study comparisons (1, 2). Substantial variability also exists in microbiome profiling methodologies. Most studies have relied on 16S rRNA gene amplicon sequencing, which provides taxonomic resolution only at the genus level and is susceptible to primer bias, whereas fewer studies have employed metagenomic sequencing, which offers species-level and functional resolution (7, 8). The choice of bioinformatic pipelines—including reference databases (Greengenes versus SILVA), clustering algorithms (OTU versus ASV), and statistical approaches—can further influence the reported microbial composition and observed associations. In addition, important confounding factors are often inadequately controlled. Maternal diet, pre-pregnancy BMI, gestational weight gain, ethnicity, socioeconomic status, antibiotic use, and mode of delivery are all known to shape the gut microbiota and may confound the observed associations with GDM (9, 25). Many studies have failed to collect or adjust for these variables, raising the possibility of residual confounding. Finally, the majority of studies are cross-sectional or prospective observational in design, which limits the ability to infer causality. Future studies should adopt standardized protocols for GDM diagnosis, sample collection, sequencing, and data analysis, and should prospectively collect comprehensive covariate data to minimize bias and enhance reproducibility.

## 7 Conclusion

This review consolidates evidence that maternal gut microbiota dysbiosis serves as both a key initiating factor and a perpetuator of the chronic low-grade inflammation that drives insulin resistance in GDM. This conclusion is supported by converging evidence from observational, animal, and preliminary interventional studies. The bidirectional gut microbiota–inflammation axis—wherein dysbiosis promotes inflammation, which in turn exacerbates dysbiosis—constitutes a self-reinforcing cycle that not only underlies maternal metabolic deterioration but also programs long-term metabolic and neurodevelopmental risks in offspring through transgenerational mechanisms.

It is important to emphasize that the majority of human studies reviewed herein are observational in nature, and thus the reported associations between gut microbiota composition and GDM do not necessarily imply causation. While animal models and fecal microbiota transplantation experiments provide stronger evidence for a causal role, direct causal inference in humans remains limited. Well-designed randomized controlled trials and Mendelian randomization studies are needed to establish whether specific microbial alterations are causative or merely correlative in GDM pathogenesis.

The central message of this manuscript is that the gut microbiota represents a modifiable and promising therapeutic target for GDM. However, the field must move beyond one-size-fits-all probiotic approaches toward stratified, microbiome-guided strategies. Future research should focus on establishing causal relationships between specific microbial strains and metabolic outcomes, identifying optimal intervention windows, and rigorously evaluating long-term health outcomes for both mother and child. By integrating mechanistic insights with personalized nutritional and microbial interventions, we can harness the gut microbiota to improve metabolic health across two generations.
